# Breast density and the likelihood of malignant MRI-detected lesions in women diagnosed with breast cancer

**DOI:** 10.1007/s00330-023-10072-w

**Published:** 2023-08-30

**Authors:** Antti Sassi, Annukka Salminen, Arja Jukkola, Maija Tervo, Niina Mäenpää, Saara Turtiainen, Leena Tiainen, Timo Liimatainen, Teemu Tolonen, Heini Huhtala, Irina Rinta-Kiikka, Otso Arponen

**Affiliations:** 1 Department of Radiology, Tampere University Hospital, Elämänaukio 1, 33520 Tampere, Finland; 2 Faculty of Medicine and Health Technology, Tampere University, Tampere, Finland; 3 Department of Oncology, Tays Cancer Center, Tampere University Hospital, Tampere, Finland; 4 Department of Surgery, Tampere University Hospital, Tampere, Finland; 5 Research Unit of Medical Imaging Physics and Technology, University of Oulu, Oulu, Finland; 6 Department of Radiology, Oulu University Hospital, Oulu, Finland; 7 Department of Pathology, Fimlab Laboratories, Tampere University Hospital, Tampere, Finland; 8 Faculty of Social Sciences, Tampere University, Tampere, Finland

**Keywords:** Breast neoplasms, Breast density, Magnetic resonance imaging, Incidental findings

## Abstract

**Objectives:**

To assess whether mammographic breast density in women diagnosed with breast cancer correlates with the total number of incidental magnetic resonance imaging (MRI)-detected lesions and the likelihood of the lesions being malignant.

**Methods:**

Patients diagnosed with breast cancer meeting the EUSOBI and EUSOMA criteria for preoperative breast MRI routinely undergo mammography and ultrasound before MRI at our institution. Incidental suspicious breast lesions detected in MRI are biopsied. We included patients diagnosed with invasive breast cancers between 2014 and 2019 who underwent preoperative breast MRI. One reader retrospectively determined breast density categories according to the 5^th^ edition of the BI-RADS lexicon.

**Results:**

Of 946 patients with 973 malignant primary breast tumors, 166 (17.5%) had a total of 175 (18.0%) incidental MRI-detected lesions (82 (46.9%) malignant and 93 (53.1%) benign). High breast density according to BI-RADS was associated with higher incidence of all incidental enhancing lesions in preoperative breast MRIs: 2.66 (95% confidence interval: 1.03–6.86) higher for BI-RADS density category B, 2.68 (1.04–6.92) for category C, and 3.67 (1.36–9.93) for category D compared to category A (*p* < 0.05). However, high breast density did not predict higher incidence of malignant incidental lesions (*p* = 0.741). Incidental MRI-detected lesions in the contralateral breast were more likely benign (*p* < 0.001): 18 (27.3%)/48 (72.7%) vs. 64 (58.7%)/45 (41.3%) malignant/benign incidental lesions in contralateral vs. ipsilateral breasts.

**Conclusion:**

Women diagnosed with breast cancer who have dense breasts have more incidental MRI-detected lesions, but higher breast density does not translate to increased likelihood of malignant incidental lesions.

**Clinical relevance statement:**

Dense breasts should not be considered as an indication for preoperative breast MRI in women diagnosed with breast cancer.

**Key Points:**

*• The role of preoperative MRI of patients with dense breasts diagnosed with breast cancer is under debate.*

*• Women with denser breasts have a higher incidence of all MRI-detected incidental breast lesions, but the incidence of malignant MRI-detected incidental lesions is not higher than in women with fatty breasts.*

*• High breast density alone should not indicate preoperative breast MRI.*

**Supplementary Information:**

The online version contains supplementary material available at 10.1007/s00330-023-10072-w.

## Introduction

Breast cancer is the most frequently diagnosed new cancer in women globally [1]. Although mammography and ultrasound are the primary imaging modalities in breast cancer diagnostics, preoperative breast magnetic resonance imaging (MRI) has gained a growing role in preoperative evaluation [2, 3]. Breast MRI offers high overall sensitivity with reported moderate pooled specificity (52–75%) in detecting breast cancer [4–6]. Breast MRI also offers high sensitivity in detection of additional lesions that are occult in clinical examination, mammography, and ultrasound [7–10]. The prevalence of incidental MRI-detected lesions has been reported to range between 11 and 29% [11–15]. Currently, the predisposing factors to incidental malignant lesions remain unknown.

The association between high breast density and elevated breast cancer risk is well documented [16]. High breast density lowers the sensitivity of mammography [17, 18], and it has been proposed that supplemental MRI screening might benefit women with extremely dense breasts [19]. Although no evidence supports the use of preoperative breast MRI in any indication from the perspective of improved disease-free or overall survival [14, 20], current European oncological and radiological guidelines [21, 22] and American oncological guidelines [23] suggest considering preoperative breast MRI for patients diagnosed with breast cancer who have mammographically dense breasts.

It remains to be established whether women with dense breasts have more incidental MRI-detected breast lesions and higher yield of malignant lesions than women with non-dense breasts. In a large registry-based study published in 2022, Onega et al [23] showed that women with dense breasts who undergo preoperative MRI have higher biopsy rates without a concomitant higher rate of MRI-detected incidental malignant lesions. Onega et al were not able to attribute the results conclusively to MRI due to the registry-based nature of their study [23]. Furthermore, a cohort study by Elmi et al [15] with 388 patients (201 imaged with digital mammography and 187 with tomosynthesis) suggested that the ability of MRI to detect additional malignancies was similar in non-dense and dense breasts.

Our aim was to evaluate whether mammographic breast density associates with (1) the total number of incidental lesions and (2) the number of malignant incidental lesions detected in MRI in a large cohort of women who underwent mammography and ultrasound imaging prior to preoperative MRI. We also evaluated the influence of laterality and histological types of primary cancer on the incidence and malignancy of MRI-detected lesions.

## Materials and methods

### Study population

From 2014 to 2019, approximately 3300 women were diagnosed with breast cancer at Tampere University Hospital (catchment area of 530,000 people). We retrospectively reviewed all patients who underwent preoperative breast MRI examinations during this period. The study was approved and the need for patients’ consent was waived with research permission granted by the Institutional Review Board of Tampere University Hospital (study code: R19627S) in accordance with the national laws and regulations.

In our hospital, patients suspected of having a breast cancer undergo bilateral breast mammograms and an ultrasound examination of the breasts and axillae according to the European guideline [21]. Indications for preoperative staging of patients with invasive breast cancer undergoing breast MRI during the study period were (1) poor demarcation of primary tumor on mammography, (2) breast tumor with lobular histology, (3) biopsy-confirmed axillary metastasis without known primary tumor, (4) suspicion of multifocal disease based on mammography or ultrasound if breast-conserving surgery was planned, (5) inconclusive findings on conventional breast imaging, and (6) known genetic predisposition to breast cancer. However, patients with genetic predisposition to breast cancer who underwent breast MRI only as part of the screening protocol were not included. The indications used to refer women with invasive cancer to preoperative breast MRI conform to the major current treatment guidelines [2, 3, 21, 22, 24, 25]. Incidental MRI-detected lesions deemed not benign (i.e., Breast Imaging Reporting & Data System (BI-RADS) 3, 4, and 5 lesions) are routinely biopsied preoperatively in our institution to avoid the need for radiological follow-up.

**Fig. 1:**
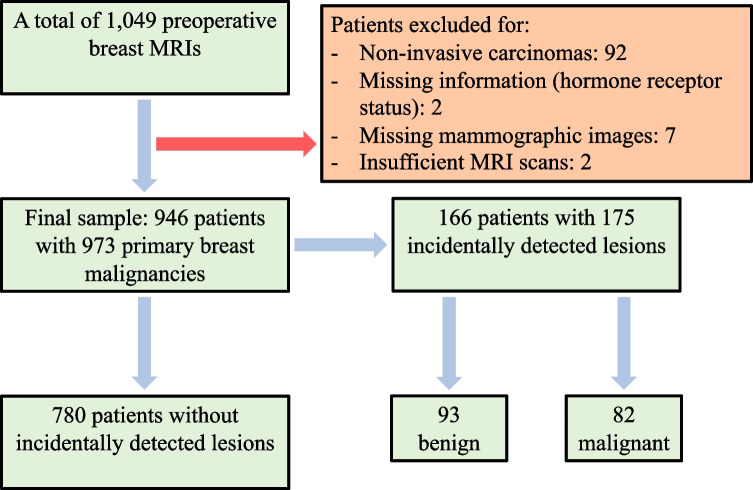
Flowchart describing the patient selection

We included women who fulfilled the following inclusion criteria: (1) MRI performed after bilateral mammography and ultrasound and (2) histopathologically confirmed invasive primary breast cancer (i.e., patients with non-invasive primary breast cancers were excluded). We excluded patients with ductal carcinoma in situ (DCIS), as we expected the rate of incidental MRI-detected lesions to be lower among patients with DCIS than among those with invasive carcinoma. Indeed, Keymeulen et al [26] reported that only 19% of the women with the diagnosis of DCIS in their sample underwent breast MRI. Of those who underwent MRI, 0.8% had a contralateral DCIS and 1.3% had a contralateral invasive carcinoma not detected on mammography. Women with missing hormone receptor parameters, insufficient mammographic images, or insufficient MRI scans were excluded from this study (Fig. 1).

### Data collection

All radiological reports were manually reviewed by two readers: Ai.S. (radiology consultant with 4 years of expertise in breast radiology) and M.T. (medical degree student). Information regarding the patients’ radiological findings in respect to the presence of incidental MRI-detected lesions was recorded from the Picture Archiving and Communication System. Patient characteristics, including the histopathology of the primary tumors and incidental lesions, were collected from the hospital’s electronic database.

### MRI protocol

The women were imaged with either 1.5-T (Siemens Magnetom Aera, Siemens AG) or 3.0-T (Philips Ingenia, Philips Healthcare Company) scanners using dedicated breast coils during the study period. T1- and T2-weighted and dynamic contrast-enhanced sequences were imaged. Routine use of diffusion-weighted imaging (DWI) sequences was introduced in August 2019, but DWI was only added as part of a different study and was not used to characterize incidental lesions during the study period. Details of the MRI sequences are provided in Supplementary materials. The MRI examinations were performed in the prone position with dedicated breast coils. A fixed dose of 14 ml gadoteric acid (Dotarem 279.3 mg/ml, Guerbet S) was used as an intravenous MRI contrast agent.

### Breast density evaluation and determination of the location of the incidental MRI-detected lesion

**Fig. 2:**
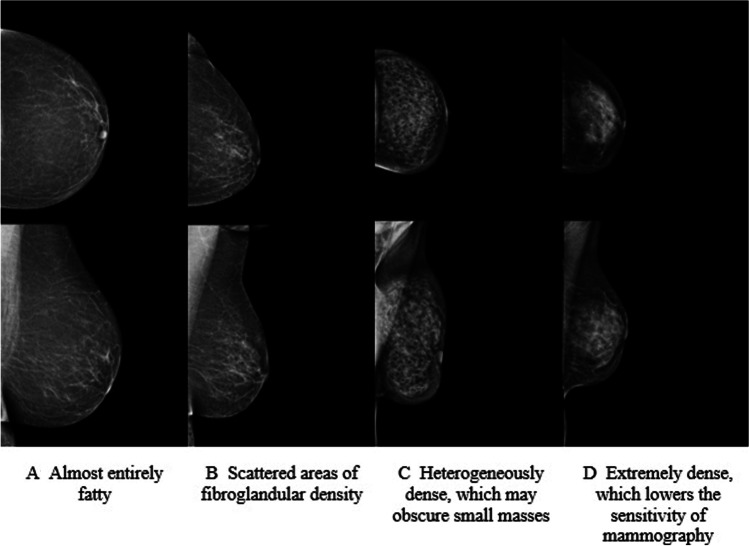
BI-RADS 5^th^ edition breast density categories

Mammographic breast densities were retrospectively divided into four categories according to the 5^th^ edition of the BI-RADS lexicon [27] (Fig. 2) from digital full-field mammograms by an experienced breast radiologist (Aa.S.) with 8 years of experience in breast radiology. The reader was blinded to the presence and the final histopathology of the incidental MRI-detected lesions.

**Fig. 3:**
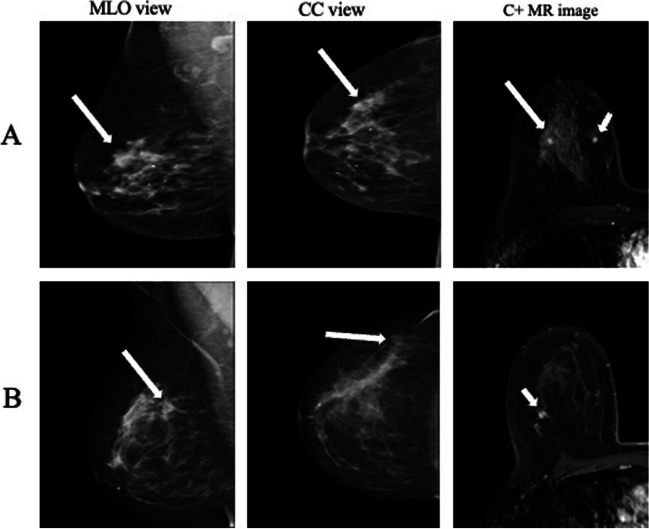
Incidental MRI-detected lesions not visible on bilateral mammograms or ultrasound were biopsied if they were BI-RADS 3–5 lesions. Two cases of incidental lesions (a fibroadenoma of 5 mm (**A**) and a carcinoma of no special type of 7 mm (**B**)) detected in women in their mid-60s with breast cancer were not detected prior to MRI on either mammography or ultrasound. Long arrows point to the primary tumor and short arrows to the incidental MRI-detected lesion. MLO, mediolateral oblique; CC, craniocaudal; C+, contrast enhanced

MRI images were reviewed by a radiology consultant (Ai.S.) for conformation of the incidental MRI-detected lesions’ location, which was recorded as follows: (1) lesion in the same breast quadrant as the primary tumor, (2) lesion in a different quadrant but in the same breast as the primary tumor, and (3) lesion in the contralateral breast. If the patient had known bilateral breast cancer prior to the breast MRI, the incidental lesion was located according to the disease in the ipsilateral breast (Fig. 3)*.*

### Evaluation of histopathological samples

Primary tumors and incidental MRI-detected lesions were biopsied under either ultrasound guidance (14G core needles) or MRI guidance (10G vacuum needles). In the case of BI-RADS 3–5 incidental MRI-detected lesions, ultrasound-guided core needle biopsy was performed immediately after breast MRI; if the lesion required MRI-guided biopsy or if the histopathology of the biopsied lesion was discordant with the imaging findings, the lesion was (re-)biopsied within 3 weeks after MRI.

Core needle and surgical samples were evaluated by in-house breast pathologists. Radio-histopathological review of the biopsied lesions was carried out in multidisciplinary breast meetings that occur twice a week to ensure that representative samples were obtained. Incidental MRI-detected lesions were classified as benign or malignant (both invasive and non-invasive malignant tumors) based on their histopathology.

### Statistical analysis

The data were analyzed with SPSS (IBM SPSS Statistics for Windows, Version 27.0, IBM Corp.). Nominal values are presented as absolute values and percentages. Continuous variables are expressed as means and standard deviations unless otherwise stated. Chi-square or Fisher’s exact tests were used to investigate the association between nominal variables based on the sizes of the groups. Binary logistic regression analysis was used to evaluate whether the breast density, laterality of the incidental lesion in relation to the primary tumor, or the histopathological subtype of the primary tumor is associated with the presence of MRI-detected incidental or malignant incidental lesions. Odds ratios are presented only if the finding is statistically significant. The analyses (including the logistic regression analyses) were performed on a breast level (i.e., the two breasts were included separately in the analysis when the patient had bilateral disease known prior to breast MRI) unless otherwise stated. A *p*-value of 0.05 or less was considered to indicate statistical significance.

## Results

### Study populations

During the study period, 1049 patients with malignant breast tumors (non-invasive and invasive) underwent preoperative breast MRI after mammography and ultrasound. Ninety-two patients (8.8%), reflecting the institutional indications for MRI, had non-invasive ductal carcinoma in situ and were thus excluded; the remaining 957 (91.2%) patients had invasive breast tumors (Fig. 1). Prior to preoperative breast MRI, 30 patients (2.9%) were known to have bilateral breast cancers. Of patients with invasive primary cancers, two were excluded due to incomplete pathological results, seven due to missing mammographic images, and two due to insufficient MRI scans. In summary, 946 patients (mean age 59.75 years, SD 11.75 years, age range 29–87 years) with a total of 973 primary breast malignancies (bilateral cancers, *n* = 27, considered as independent diseases) were included (Fig. 1). Patient characteristics are presented in Table 1.

**Table 1:** Characteristics of all patients and those diagnosed with either malignant or benign incidental MRI-detected lesions or only malignant incidental MRI-detected lesions

	All patients *N* = 946		Patients with at least one incidental lesion^a^ *n* = 166		Patients with at least one malignant incidental lesion *n* = 77	
	*n*	%	*n*	%	*n*	%
Age (mean (years), *SD*, min–max)	59.75, *11.75*, 29–87		59.14, *11.85*, 31–85		59.14, *12.19*, 34–85	
Primary tumor histology						
Invasive cancer of no special type	672	71.0	118	71.1	52	67.5
Lobular	223	23.6	39	23.5	22	28.6
Other	51	5.4	9	5.4	3	3.9
Intrinsic subtype^b^						
Luminal A	322	34.0	67	40.4	36	46.8
Luminal B	515	54.4	84	50.6	35	45.5
Basal-like	63	6.7	8	4.8	2	2.6
HER2-enriched	46	4.9	7	4.2	4	5.2
Breast density						
A	66	7.0	4	2.4	3	3.9
B	384	40.6	67	40.4	31	40.3
C	360	38.1	64	38.6	29	37.7
D	136	14.4	31	18.7	14	18.2

^a^BI-RADS category 3–5 lesions

^b^Cutoff value of ≥ 1% was used to define positive hormone receptor status (estrogen and progesterone)

### Incidence of MRI-detected lesions and their prevalence in density groups

Among the 946 women with known breast cancers, 166 (17.5%) were recommended for biopsies of 175 MRI-detected BI-RADS 3–5 lesions. The biopsies confirmed 93 (53.1%) of these lesions as benign and 82 (46.9%) as malignant (both invasive and non-invasive malignant incidental lesions). None of the histological types of the primary invasive cancer increased the incidence of all incidental lesions (*p* = 0.972) or malignant incidental lesions (*p* = 0.305; Table 2). Furthermore, the laterality of biopsied lesions was not associated with the incidence of (1) all MRI-detected lesions (*p* = 0.955) or (2) only malignant lesions (*p* = 0.083) in any histological groups of primary cancer (Table 3). The location of the lesion in the ipsilateral breast was significantly associated with the malignity of the lesion (64 (58.7%) malignant in ipsilateral breast, 18 (27.3%) malignant in contralateral breast (*p* < 0.001)). Benign incidental lesions were equally distributed into ipsilateral and contralateral breasts: 45 (48.4%) ipsilateral vs. 48 (51.6%) contralateral. A total of 116 (12.3%) women (with 119 known cancers) were imaged with a 1.5-T scanner and 830 (87.7%; with 854 known cancers) with a 3.0-T scanner. Those scanned with a 1.5-T scanner had fewer incidental MRI-detected biopsy-requiring lesions and malignant MRI-detected incidental lesions: 10 (8.4%) and 4 (3.4%) vs. 158 (18.5%) and 75 (8.8%), respectively (*p* = 0.006/0.043 for all and malignant MRI-detected incidental lesions).

**Table 2:** Incidence of all MRI-detected lesions (*n* = 175) and only malignant MRI-detected lesions (*n* = 82) in different histological subtypes of the primary breast cancer

	Primary carcinoma						
	No special type *n* = 695		Lobular *n* = 234		Other *n* = 51		*p*-value
	*n*	%	*n*	%	*n*	%	
All incidental MRI-detected lesions^a^	123	17.7	43	18.4	9	17.6	0.972
Malignant MRI-detected incidental lesions	54	7.8	25	10.7	3	5.9	0.305

^a^BI-RADS category 3–5 lesions

**Table 3:** Distribution of laterality of all incidental lesions and only malignant incidental lesions in different histological subtypes of the primary breast cancer. Numbers represent the total numbers of all and malignant incidental lesions in ipsilateral/contralateral breasts, respectively. Percentage share indicates the proportion of all contralateral and malignant contralateral incidental MRI-detected lesions

	Primary carcinoma						
	No special type		Lobular		Other		*p*-value
	*n*^a^	%^b^	*n*^a^	%^b^	*n*^a^	%^b^	
All incidental MRI-detected lesions^c^	76/47	38.2	27/16	37.2	6/3	33.3	0.955
Malignant incidental MRI-detected lesions	43/11	20.4	19/6	24.0	2/1	33.3	0.832

^a^Ipsilateral/contralateral side

^b^Percentage share of contralateral incidental MRI-detected lesions

^c^BI-RADS category 3–5 lesions

**Fig. 4:**
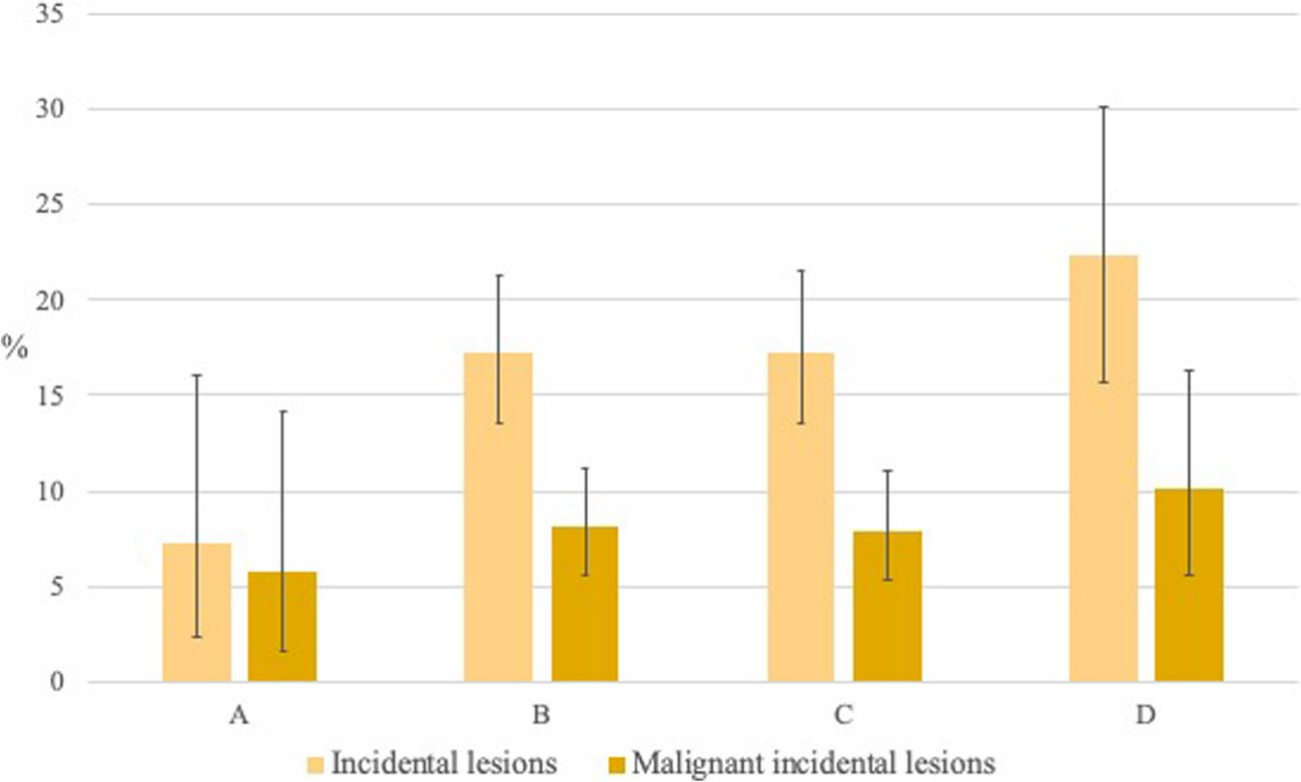
Incidence of all incidental lesions and malignant incidental lesions in BI-RADS 5^th^ edition breast density categories (*n* = 973). Whiskers indicate 95% confidence intervals

Sixty-six (7.0%) patients were categorized as having BI-RADS density A, 384 (40.6%) BI-RADS density B, 360 (38.1%) BI-RADS density C, and 136 (14.4%) BI-RADS density D. The incidence of benign and malignant incidental MRI-detected lesions was not significantly higher in denser breasts according to the Chi-square test (*p* = 0.062). However, the odds ratio for either benign or malignant incidental lesions was 2.66 (95% confidence interval (CI): 1.03–6.86) higher for BI-RADS density category B, 2.68 (1.04–6.92) for category C, and 3.67 (1.36–9.93) for category D in comparison to density category A (*p* < 0.05; Table 1 and Fig. 4). Higher breast density was not significantly associated with the number of malignant incidental lesions (*p* = 0.741) and did not statistically increase the risk of being diagnosed with incidental MRI-detected malignant breast lesions (Fig. 4). The odds ratio for malignant incidental lesions in the breast was 1.43 (95% CI: 0.49–4.19) for BI-RADS density category B, 1.38 for category C (0.47–4.06), and 1.82 for category D (95% CI: 0.58–5.75) in comparison to density category A (*p* = 0.744; Fig. 4). When breast density was dichotomized as high (BI-RADS density categories C+D) and low (BI-RADS density categories A+B), no significant differences were observed in the incidence of all (95 (18.7%) high vs. 73 (15.7%) low, *p* = 0.227) or malignant (43 (8.4%) high vs. 36 (7.8%) low, *p* = 0.694) incidental MRI-detected lesions.

## Discussion

In our sample, approximately one in five women undergoing preoperative breast imaging had incidental MRI-detected lesions. Approximately half of the incidental lesions were malignant. Our main finding is that women with denser breasts had a higher prevalence of incidentally detected breast lesions, but they did not have more malignant incidentally detected breast lesions. We conclude that higher breast density alone should not indicate preoperative breast MRI, as malignant MRI-detected lesions are not statistically more common in women with dense breasts. Using dense breasts as an indication for preoperative MRI would increase the woman’s risk of undergoing unnecessary biopsy for benign incidental MRI-detected lesions without the added benefit in detecting more malignant breast lesions. Furthermore, the histopathological subtype of the primary cancer did not predict the laterality or malignancy of the MRI-detected incidental lesions, but the ipsilateral MRI-detected lesions were more commonly malignant than contralateral MRI-detected lesions.

High breast density has been associated with elevated breast cancer risk [16, 28, 29]. Furthermore, high breast density decreases the sensitivity of digital mammography [17, 18]. Current diagnostic guidelines for breast cancer treatment suggest considering preoperative breast MRI for women with breast cancer who have dense breasts [21–23]. Our study is, we believe, the first one to show that women with dense breasts have more incidental findings but are not at greater risk of being diagnosed with malignant incidental MRI-detected lesions. Our conclusions are supported by Onega et al [23], who in 2022 showed with a registry-based sample of 19,324 women that women with dense breasts who undergo preoperative MRI have higher biopsy rates without a concomitant higher rate of malignant lesions. However, Onega et al were not able to attribute the results conclusively to MRI due to the registry-based nature of their study [23]. Importantly, the rate of incidental MRI-detected breast lesions and malignant incidental MRI-detected breast lesions in our study (17.5% and 8.7%, respectively) is in line with previous studies that have reported the prevalence of incidental and malignant incidental lesions to range between 10.9 and 29.1% and between 4.2 and 12.9%, respectively [11–13, 15, 23].

Given the high malignancy rate of MRI-detected lesions, incidental MRI-detected lesions cannot be ignored. The literature suggests that the BI-RADS-based morphological and kinetic features do not accurately rule out malignancy of incidental lesions [8, 14]. DWI has been proposed as a potential method to differentiate benign and malignant lesions [7, 30]. However, although DWI can reduce the number of unnecessary biopsies (i.e., false positive findings) by 20.9% without affecting sensitivity in the diagnostics of primary breast tumors [30], it cannot rule out malignancy of all incidental MRI-detected lesions. Most incidental MRI-detected lesions still warrant histopathological sampling. Indeed, the importance of our finding and the significance of the continuous evaluation of MRI indications are underlined by the fact that the MRI-detected lesions warrant further evaluation because they cannot, with confidence, be deemed benign based on their imaging features.

Our study has several strengths. Despite being a retrospective single-center study, it is one of the largest published. Furthermore, all patients underwent both mammographic and ultrasound examinations prior to MRI; indications for preoperative breast MRI in our institution adhered to the international guidelines [2, 22]; and, according to the local practice, all BI-RADS 3–5 lesions were biopsied after MRI. Furthermore, breast density was evaluated by one reader blinded to the MRI findings. However, the study also has limitations. Density estimation was performed by one experienced breast radiologist. An automated density estimation tool could help to improve the generalizability of the study. We chose to analyze images visually to study the effect of the breast density categories according to the 5^th^ edition of BI-RADS. Raw images were not available for all the patients and the commercially available automatic density evaluation tools, such as Volpara (Volpara Solutions) and Densitas (Densitas Inc.) [31], were therefore not of use. In addition, we did not study the effect of breast density on preoperative mammographic, sonographic, and MRI evaluation of tumor size. In the future, multicenter studies are needed to evaluate the criteria for preoperative breast MRI. Finally, we encourage further efforts, including development and testing of novel sequences, to rule out malignancy of incidental MRI-detected lesions with greater confidence.

In conclusion, we found that women with dense breasts have a higher overall incidence of incidental MRI-detected breast lesions than women with non-dense breasts. However, increased breast density did not associate with statistically higher incidence of malignant incidental lesions. We conclude that breast density alone should not be considered as an indication for preoperative breast MRI.

### Supplementary Information

ESM 1 (PDF 147 kb)

## Abbreviations

BI-RADS: Breast Imaging Reporting & Data System
DCIS: Ductal carcinoma in situ
DWI: Diffusion-weighted imaging
MRI: Magnetic resonance imaging

## Declarations

### Guarantor

The scientific guarantor of this publication is Adjunct Professor Irina Rinta-Kiikka, Tampere University Hospital.

### Statistics and biometry

One of the authors, Heini Huhtanen, has significant statistical expertise.

### Methodology

- Retrospective
- Cross-sectional study
- Performed at one institution
