# Supplementary material for: Breast density and the likelihood of malignant MRI-detected lesions in women diagnosed with breast cancer
Source: Eur Radiol. 2023 Aug 30;33(11):8080–8. doi: 10.1007/s00330-023-10072-w (PMC10598189; doi:10.1007/s00330-023-10072-w)
Supplement: Supplementary file 1 — (PDF 147 kb) [file 330_2023_10072_MOESM1_ESM.pdf]

Breast density and the likelihood of malignant MRI-detected lesions in women diagnosed with breast cancer

Electronic Supplementary Material

Imaging protocol

The imaging protocol used for the 1.5T scanner consisted of the following sequences: (1) T1-weighted 3D FLASH NFS (TR 8.8 ms, TE 4.77 ms, in-plane resolution 0.8×0.8 mm, slice thickness 1.5 mm, flip angle 20°), (2) T2-weighted TSE (TR 5,000 ms, TE 86 ms, in-plane resolution 0.5×0.5 mm, slice thickness 3 mm, flip angle 180°), (3) T2-weighted TIRM fat-saturated sequence (TR 4,000 ms, TE 57 ms, in-plane resolution 0.8×0.8 mm, slice thickness 4 mm, flip angle 170°), and (4) dynamic contrast T1-weighted 3D dynaVIEWs (TR 4.5 ms, TE 5.22 ms, in-plane resolution 0.8×0.8 mm, slice thickness 2 mm, flip angle 90°) and delayed views T1-weighted FS (TR 11.1 ms, TE 5.22 ms, in-plane resolution 0.8×0.8 mm, slice thickness 2 mm, flip angle 90°).

The imaging protocol used for the 3.0T scanner consisted of the following sequences: (1) T1-weighted 3D fast field echo (TR 4.82 ms, TE 2.4 ms, in-plane resolution 0.9×0.9 mm, slice thickness 1.8 mm, flip angle 10°), (2) T2-weighted TSE (TR 4,380 ms, TE 120 ms, in-plane resolution 1.0×0.8 mm, slice thickness 1.8 mm, flip angle 90°), (3) T2-weighted STIR fat-saturated sequence (TR 4,767 ms, TE 65 ms, in-plane resolution 1.0×1.0 mm, slice thickness 3 mm, flip angle 90°), (4) DWI SPAIR (in-plane resolution 2.0×2.0 mm, slice thickness 3 mm, b-values of 0, 20, 50, 100, 200, 850, 1,000, 1,400 s/mm^2) (introduced in August 2019), and (5) T1-weighted SPAIR Dynamic eThrive (TR 4.82 ms, TE 2.4 ms, in-plane resolution 1.0×0.9 mm, slice thickness 1.8 mm, flip angle 10°).
